# Intravitreal Aflibercept as an Adjunct to Systemic Therapy in a Case of Choroidal Neovascular Membrane Associated with Sympathetic Ophthalmia

**DOI:** 10.4274/tjo.09076

**Published:** 2018-09-04

**Authors:** Ali Osman Saatçi, Ziya Ayhan, Şefik Can İpek, Meltem Söylev Bajin

**Affiliations:** 1 Dokuz Eylül University Faculty of Medicine, Department of Ophthalmology, İzmir, Turkey

**Keywords:** Aflibercept, choroidal neovascular membrane, prednisolone, sympathetic ophthalmia

## Abstract

Choroidal neovascular membrane (CNV) is a very rare complication in patients with sympathetic ophthalmia. We hereby report a 38-year-old man who developed a type 2 CNV in his only seeing (left) eye while under systemic steroid treatment. Systemic therapy was revised and a total of 5 intravitreal aflibercept injections (2 mg each) were administered over a period of 8 months. Good anatomic and functional outcome was noted at the last visit. Anti-vascular endothelial growth factor injection may be an important adjunct to systemic therapy in eyes with with sympathetic ophthalmia-associated CNV.

## Introduction

Sympathetic ophthalmia (SO) is a rare bilateral granulomatous inflammation that follows an accidental or surgical insult to the uvea.^1^ Choroidal neovascular membrane (CNV) may complicate the disease course and negatively affect visual outcome in patients with SO.^2,3,4,5,6,7,8,9^

In a retrospective cohort study including 4041 eyes of 2307 patients with posterior uveitis and panuveitis, 81 eyes (2%) had CNV at presentation.^10^ In this study, the number of patients with SO and CNV was not given as it was too small. Recently, in another retrospective study involving 6850 uveitis patients, 73 eyes of 60 patients (0.87%) were found to have inflammatory CNV.^11^ Of these 73 eyes, 52% (38 eyes of 29 patients) had CNV at the first presentation and the remaining 48% (35 eyes of 31 patients) developed CNV during the course of follow-up. Only 1 patient with CNV had a diagnosis of SO.

Here we report a one-eyed patient with SO who developed CNV during follow-up and was treated with intravitreal aflibercept injections in addition to ongoing systemic therapy.

## Case Report

**Figure 1:**
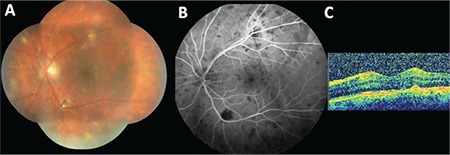
A) Composite color fundus photograph of the left eye showing scattered roundish yellow-white infiltrates throughout the fundus; B) venous phase of the angiogram depicting the widespread hypofluorescent dots under the retina in 360 degrees; C) optical coherence tomography section showing the nearly normal foveolar contour and some hyperreflective dots corresponding to posterior vitreous inflammatory cell clusters at the time of the diagnosis of sympathetic ophthalmia

**Figure 2:**
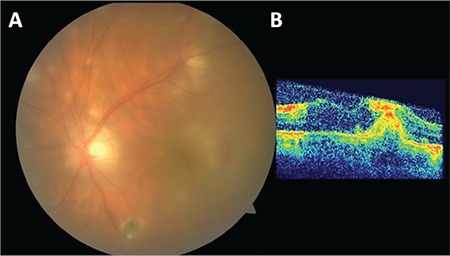
A) Color fundus photograph of the left eye demonstrating the hazy view of the posterior fundus and yellow-white foveolar infiltration with indistinct borders and the presence of some retinal hemorrhage; B) optical coherence tomography section of the left eye delineating the possible choroidal neovascular membrane

**Figure 3:**
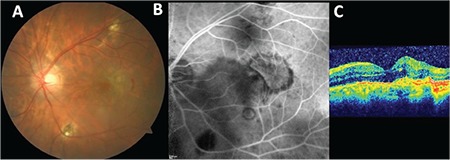
A) Color fundus photograph of the left eye showing the juxtafoveolar choroidal neovascular membrane and scattered chorioretinal scars; B) fluorescein angiography image depicting the hyperfluorescent well-demarcated classic type of choroidal neovascular membrane; C) optical coherence tomography section showing type 2 membrane with some intraretinal fluid

**Figure 4:**
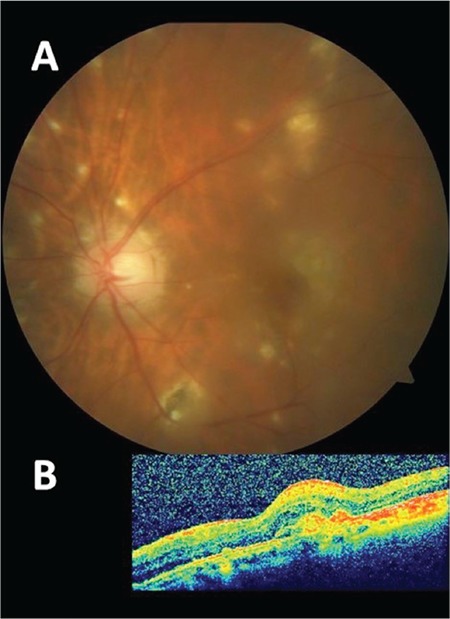
A) Color fundus photograph showing the diminished choroidal neovascular membrane area and persistent 360-degree chorioretinal scars at the last follow-up visit; B) optical coherence tomography section showing the fibrotic-looking choroidal neovascular membrane

The blind and painful right eye of a 38-year-old man was eviscerated in September 2016. The patient stated that his right eye had been blind since early childhood due to a unilateral congenital anomaly complicated by secondary glaucoma. He received the diagnosis of SO in January 2017 after he experienced visual loss in his only seeing (left) eye. At the time of diagnosis, the patient was admitted to the hospital and meticulously investigated for possible infectious and noninfectious causes to rule out other uveitic entities, but without any positive findings. At that time, his best-corrected visual acuity was 6/10. Slit-lamp examination yielded some vitreous cells in the left eye. Fundoscopy showed a few scattered pigmented chorioretinal scars and discrete yellowish round choroidal lesions throughout the left fundus (Figure 1A). Fluorescein angiogram delineated the active lesions as early hypofluorescent (Figure 1B) with late staining. Left macular contour was normal on optical coherence tomography (OCT) examination (Figure 1C). He was started on oral prednisolone (64 mg) for 2 weeks with gradual tapering of 8 mg per week. Despite initial visual improvement, he experienced another episode of visual decline while taking 32 mg of prednisolone. His best-corrected visual acuity decreased to 2/10 and he had grade 4 vitreous haze according to the Miami grading.^12^ Fundus examination showed marked yellowish-white discoloration of the macula with some evidence of intraretinal hemorrhage (Figure 2A and B). He was hospitalized and treated with pulse methylprednisolone 1 g (250 mg 4 times daily) for 3 days. Following pulse therapy, 64 mg oral prednisolone and 150 mg (50 mg 3 times daily) azathioprine were co-administered. Two weeks after the completion of pulse therapy, his visual acuity was still 2/10 despite a significant reduction in vitreous haze. Fluorescein angiogram and OCT demonstrated type 2 choroidal neovascularization (Figure 3A-C). Five intravitreal 2 mg aflibercept injections were given within a period of 8 months. His final visual acuity was 6/10 with a stable-looking macula (Figure 4A and B) and he was continued on a treatment regimen of 150 mg azathioprine and 8 mg prednisolone daily.

## Discussion

CNV can be associated with various inflammatory choroidal diseases, as cytokines together with vascular endothelial growth factor (VEGF) are implicated in the pathogenetic mechanisms, leading to impaired permeability and altered angiogenesis.^13,14^ The presence of Dalen-Fuchs nodules may be the cause of the disruption of the Bruch’s membrane in eyes with SO.^7^

CNV in eyes with SO has been observed to resolve spontaneously^2,3^ and to respond to immunosuppressive agents (cyclosporine and azathioprine),^4,7^ photodynamic therapy,^6^ thermal laser,^5^ and intravitreal bevacizumab injection.^8^ Even submacular surgery to remove CNV has been attempted.^9^

Steroids and immunosuppressives are generally used in uveitis to reduce the inflammatory stimulus which seems to play a role in CNV formation and also partly due to their antiangiogenic effect.^15^ Anti-VEGF agents have also been used in addition to systemic therapy in eyes with inflammatory CNV in case reports and series. In a randomized study investigating the efficacy and safety of ranibizumab for the treatment of CNV due to uncommon causes in a group of 178 patients, 18 (15.1%) had postinflammatory type CNV and a single loading dose followed by a pro re nata regimen seemed to succeed statistically both in anatomic and visual outcome.^16^ Julian et al.^8^ reported using intravitreal bevacizumab therapy in a group of 15 patients with uveitis-related CNV and only 1 of the 15 patients had SO. This patient was also receiving systemic steroid and interferon alpha. The number of bevacizumab injections was not mentioned. In our case, the patient developed CNV in his only eye while he was on systemic steroids and 5 consecutive 2 mg aflibercept injections administered over 8 months helped us to improve his vision. Together with azathioprine maintenance therapy, he achieved relatively good anatomic and visual outcomes.

Clinicians should be careful to differentiate the signs of choroidal subretinal neovascular membrane from the signs of already present uveal inflammation, as even eyes under immunosuppressive treatment may develop CNV. Anti-VEGF agents (aflibercept in the present case) seems to be a very valuable adjunct to obtain a good anatomic and functional outcome in inflammatory type subretinal choroidal neovascular membranes and systemic immunosuppression should also be used to suppress the inflammatory stimulus.
